# Plasma fingerprint of free fatty acids and their correlations with the traditional cardiac biomarkers in patients with type 2 diabetes complicated by coronary heart disease

**DOI:** 10.3389/fcvm.2022.903412

**Published:** 2022-07-22

**Authors:** Ting Hu, Wen Zhang, Feifei Han, Rui Zhao, Lihong Liu, Zhuoling An

**Affiliations:** Beijing Chao-Yang Hospital, Capital Medical University, Beijing, China

**Keywords:** free fatty acid, type 2 diabetes mellitus, coronary heart disease, risk factor, fingerprint

## Abstract

Type 2 diabetes mellitus (T2DM) is a well-established risk factor for cardiovascular disease, with at least 2–3 fold higher risk of cardiovascular diseases than non-diabetics. Free fatty acids (FFAs) are believed to play important roles in the occurrence of cardiovascular disease in people with T2DM. The aim of this study was to investigate the fingerprint of plasma FFAs and their correlations with the tradition risk factors of cardiovascular disease in T2DM patients complicated by coronary heart disease (CHD-T2DM). A total of 401 participants, including healthy control (HC, *n* = 143), T2DM patients (*n* = 134), and CHD-T2DM patients (*n* = 126) were enrolled in this study. Plasma levels of 36 FFAs with carbon chain length ranged from 3 to 22 were quantified by using reverse phase ultra-high performance liquid chromatography coupled with tandem mass spectrometry (UHPLC-MS/MS). Tradition risk factors of cardiovascular disease were tested in clinical laboratory, including homocysteine (HCY), creatine kinase (CK), high sensitivity C reactive protein (hsCRP), and N-terminal pro-brain natriuretic peptide (NT-proBNP) and so on. Almost all the FFAs with different carbon chain length and unsaturation were significantly upregulated in the T2DM-CHD groups, compared to the HC and T2DM groups. Both n-3 and n-6 polyunsaturated fatty acids (PUFA) were also found to be significantly upregulated in T2DM-CHD group compared to the T2DM group. However, no significantly differences of the n-6/n-3 PUFA ratio, arachidonic acid/eicosapentaenoic acid (AA/EPA) ratio, and arachidonic acid/docosahexaenoic acid (AA/DHA) ratio were observed between T2DM-CHD and T2DM groups. Plasma FFA levels were found to be positively correlated with HCY, CK, hsCRP, NT-proBNP and other tradition risk factors of CHD. Multivariate logistic regression analysis indicated that a dozens of FFAs were the independent risk factors of CHD after adjustment for confounding factors and other risk factors. Excessively high plasma levels of FFAs were demonstrated to be independent risk factors for CHD in patients with T2DM, despite of the differences in chain length, unsaturation, and double bond position.

## Introduction

Globally, the population with diabetes mellitus has quadrupled in the last 30 years (1, 2). It was estimated that 1 in 11 adults worldwide have diabetes mellitus, and 90% of them were type 2 diabetes mellitus (T2DM) (2, 3). T2DM can be attributed to insulin resistance and a corresponding failure of pancreatic islets to maintain appropriate insulin output to compensate for the decline in insulin sensitivity (2–4). T2DM is now a major chronic disease and one of the most important causes of death, which is estimated to afflict over 400 million people worldwide (2–4). And the number continues to rise due to population aging, economic development, urbanization, unhealthy eating habits and sedentary lifestyles.

Coronary heart disease (CHD) is the most common and serious complication of T2DM, accounting for about two-thirds of deaths in patients with T2DM (5, 6). T2DM is a main risk factor for CHD, independent of age, body mass index (BMI), blood pressure, and smoking status. Usually, people with T2DM are twice as likely to develop cardiovascular disease as those without T2DM (6, 7). Furthermore, poor glycemic control is associated with greater risk for the development of heart failure. It is estimated that for every 1% increase in glycated hemoglobin (HbA1c) levels, the incidence of heart failure increases by 8–36% (8). A variety of pathophysiological processes may contribute to CHD in patients with type 2 diabetes, including insulin resistance or hyperinsulinemia, dyslipidemia, chronic low-grade inflammation, oxidative stress, endothelial dysfunction, vascular calcification, and hypercoagulation (9).

Free fatty acids (FFAs), originated from adipose tissue and released by lipolysis of triglyceride and phospholipid, are the main source of myocardium (10). Epidemiological studies have demonstrated that blood FFAs levels is an independent factor for CHD (11, 12). This phenomenon is more pronounced in diabetic hearts because of their impaired glucose utilization. Excessive FFA oxidation rate promotes abnormal energy metabolism and cardiac dysfunction in diabetic myocardium (13, 14). Besides, high FFA levels cause lipid accumulation and lipotoxicity in cardiomyocytes, which contributes to systemic inflammation, oxidative stress and eventual cardiomyocyte apoptosis (15, 16). Intervention trials showed modest cardiovascular benefits by increasing intake of polyunsaturated fat while reducing intake of saturated fat (17). Long-chain polyunsaturated FFAs and their derivatives (mainly eicosanoids) regulate various inflammatory and anti-inflammatory pathways in organism (18).

The occurrence of CHD with a history of T2DM (T2DM-CHD) is the result of multiple environmental and genetic factors (19). Clinical diagnosis of T2DM-CHD is complicated. At present, blood glucose level, electrocardiogram, Doppler ultrasonography, coronary angiography and nuclear magnetic technology are all employed in the diagnosis of T2DM-CHD (5). Coronary angiography is the “gold standard” for diagnosing CHD (20). However, coronary angiography is invasive and expensive, which can impose a great burden on patients. Therefore, the exploration of new humoral biomarkers with high sensitivity and specificity can greatly reduce the trauma in the diagnosis of T2DM-CHD, which is of great significance for the diagnosis of T2DM-CHD and the improvement of its clinical outcome.

Serum indicators have been used in the auxiliary diagnosis of T2DM-CHD, including creatine kinase (CK), hypersensitive C-reactive protein (hsCRP), cardiac troponin I, lactate dehydrogenase (LDH), NT-proBNP, homocysteine, and CKMB-mass (21–23). However, most of these indicators are released into the bloodstream as a result of ischemic myocardial injury, which can hardly used for the early warning of CHD. Though many studies have explored the association between FFAs and T2DM-CHD, they mainly focused on the total serum FFA levels (24, 25). There are limited data about the roles of FFAs with different carbon chain length and unsaturation in the pathophysiology of T2DM-CHD. Therefore, the aim of the present study was to investigate the differences of plasma FFAs fingerprint among healthy controls, T2DM patients and T2DM-CHD patients. Besides, the correlations between FFAs and critical clinical indicators of cardiovascular disease were also explored.

## Materials and methods

### Chemicals and reagents

FFA standards were purchased from Sigma-Aldrich (St. Louis, MO, United States) and Cayman Chemical (Ann Arbor, MI, United States). 2-Hydrazinyl-4,6-dimethylpyrimidine (DMP) and 2-hydrazinylpyrimidine (DP) were used for the derivatization of FFAs. DMP and DP reagents were obtained from ChemBridge (San Diego, CA, United States) and J&K (Beijing, China), respectively. 2-(7-azabenzotriazol-1-yl)-N,N,N′,N′-tetramethyluronium hexafluorophosphate (HATU) and ethyldiisopropylamine (DIEA) were purchased from Sigma-Aldrich and used as condensation agents for FFA derivatization. Organic solvents of MS grade, acetonitrile and methanol, were purchased from Fisher Scientific (Pittsburgh, PA, United States). Formic acid of MS grade was acquired from Sigma-Aldrich. Ultrapure water was prepared by using a Mili-Q purification system (Bedford, MA, United States).

### Study design

Three groups of subjects were included in this study, including the healthy control (HC) group with 143 participants, the T2DM group with 134 participants, and the T2DM-CHD group with 126 participants. All the subjects were aged above 18 years old and were recruited from Beijing Chaoyang Hospital from March 2015 to April 2017. Study was conducted in accordance with the Declaration of Helsinki and was approved by the ethics committees of Beijing Chao-Yang Hospital. T2DM was diagnosed by a physician and should meet at least one of the following criteria: (1) fasting serum glucose ≥ 7.0 mmol/L; (2) oral glucose tolerance test ≥ 11.1 mmol/L, and (3) the use of diabetes medication. CHD was defined as acute myocardial infarction (MI), silent MI or having undergone coronary surgery. It was diagnosed by a cardiac surgeon according to the criteria proposed by Khera et al. (26).

The subjects in HC group were healthy people with matched age and gender to the disease groups. The HCs were free of diabetes mellitus, hypertension, cardiovascular disease, metabolic syndrome, or any related family histories. The T2DM group included 143 T2DM patients with poor glycemic control, which was defined by a glycosylated hemoglobin (HbA1c) level ≥ 6%. The subjects in T2DM group should be free of any cardiovascular events. The T2DM-CHD group included 126 patients with a definite diagnosis of both T2DM and CHD, and the history of diabetes should be longer than the history of CHD. The exclusion criteria was as follows: type 1 diabetes mellitus, pregnancy, severe liver and kidney dysfunction, psychiatric disorder, substance abuse disorder, history of malignant tumor, and any other conditions which were concerned that could interfere with the final result altering the FFA pattern.

### Blood collection and clinical laboratory testing

Fasting blood samples were collected by using a purple cap anticoagulant blood collection tube. The blood samples were centrifuged at 3,000 *g* for 10 min to obtain plasma. All the plasma samples were stored at −70°C until further FFA quantification. A face-to-face interview was conducted to collect basic demographic information of each participant, including height, weight, medication history, smoking and drinking history, pregnancy history, disease history, and family disease history. Routine blood tests, including blood biochemistry test, routine blood test, coagulation function, homocysteine, HbA1c, hsCRP, NT-proBNP, and serum myocardial zymogram, were carried out by the hospital laboratory department.

### Quantification of plasma free fatty acids by UHPLC-MS/MS

A total of 36 FFAs with carbon chain length ranged from 3 to 22 were quantified by using reverse phase ultra-high performance liquid chromatography coupled with tandem mass spectrometry (UHPLC-MS/MS). Quantification was conducted according to a previously published method by our laboratory (27). An API 5500 mass spectrometer (AB Sciex, Canada) coupled with a Spark Holland liquid chromatograph (Spark, Holland) was employed for data acquisition. A Waters BEH C18 column (100 × 2.1 mm^2^, 1.7 μm) was used for chromatographic separation. The column temperature was set at 55°C. The injection volume was 5 μL. Elution solvents were water and acetonitrile, both containing 0.1% formic acid. The flow rate was 0.5 mL/min. More detailed parameters regarding FFA quantification were published in our previous work (18).

### Data processing and statistical analysis

MultiQuant software (version 3.0.2, AB Sciex, Canada) was used for the processing of raw data. Calibration curve for each FFA was constructed by using the least-squares method with a *1/x^2^* weighting factor. SIMCA 14.1 (Umetrics AB, Umeå, Sweden) was employed for multivariate statistical analysis, including principle component analysis (PCA) and orthogonal partial least squares discriminant analysis (OPLS-DA). The quality of each OPLS-DA model was evaluated using R2 (cum) values, which identify the variations described by all components in the model. Q2 (cum) was a value calculated from sevenfold cross-validation, which represented the predictability of the modeling (28). Besides, the permutation test was used to investigate whether the OPLS-DA model was overfitted. The variable importance in projection (VIP) is the most important parameter for the evaluation of each variable in the OPLS-DA model, with VIP > 1 considered to be an important contribution to the classification model. IBM SPSS 21 (Armonk, NY, United States) was used for hypothesis testing, Spearman’s correlation and binary logistic regression analysis (backward stepwise: Wald), with *P* < 0.05 considered as statistically significant. The correlation networks of metabolic and clinical phenotypes were generated by Cytoscape 3.5.0. The correlation coefficients obtained from Spearman’s rank correlation analysis were imported to the Cytoscape 3.5.0 to generate the correlation networks. GraphPad Prim 7 was used for receiver operating characteristic (ROC) and histogram analysis.

## Results

### Demographic and baseline characteristics

The demographic and baseline characteristic of HC, T2DM and T2DM-CHD groups were listed in Table 1. No significant differences of age and gender were observed among the three groups. The T2DM-CHD group possessed the highest BMI value among the three groups, which was significantly higher than the HC group. However, no significant differences of BMI values were observed between the HC group and the T2DM group, nor was the T2DM group and the T2DM-CHD group. The smoking rate and drinking rate of the disease groups were higher than those of the HC group, and the smoking rate of the T2DM-CHD group was significantly higher than that of the T2DM group. Approximately 3% of the subjects in T2DM group were taking lipid-lowering drugs, and this number reached 10% in T2DM-CHD group. More than 95% of the subjects in both T2DM and T2DM-CHD groups were taking hypoglycemic drugs. Seventy-six percent of subjects in the T2DM-CHD group were taking hypotensive drugs, which was significantly higher than the proportion in the T2DM group. The average duration of diabetes in the disease groups were greater than 10 years, and there was no significant difference between T2DM and T2DM-CHD groups. Family history was also collected for each subject, with result listed in Table 1. Generally, family history of diabetes was more common in subjects of the T2DM group, while family history of hypertension or CHD was more common in subjects of the T2DM-CHD group.

**TABLE 1:** Demographic and clinical characteristics of the participants.

Characteristics	HC	T2DM	T2DM-CHD	*P*_1_ value (HC *vs* T2DM)	*P*_2_ value (HC *vs* T2DM-CHD)	*P*_3_ value (DM *vs* T2DM-CHD)
No. of Subjects	143	134	126			
Age (years)	60.3 ± 10.1	58.8 ± 10.1	60.8 ± 10.7	0.078	0.852	0.124
Gender				0.370	0.708	0.217
*Male*	98 (69)	85 (64)	89 (71)			
*Female*	45 (31)	49 (36)	37 (29)			
BMI (kg/m^2^)	24.8 ± 3.8	25.2 ± 3.7	26.2 ± 3.7	0.273	0.005	0.078
Smoking				1.90E-06	7.62E-13	0.009
*Yes*	22 (15)	55 (41)	72 (57)			
*No*	121 (85)	79 (59)	54 (43)			
Drinking				4.08E-07	4.79E-08	0.636
*Yes*	10 (7)	41 (31)	42 (33)			
*No*	133 (93)	93 (69)	84 (67)			
Lipid-lowering drugs						0.017
*Yes*	0 (0)	4 (3)	13 (10)			
*No*	143 (100)	130 (97)	113 (90)			
Hypoglycemic drugs						0.303
*Yes*	0 (0)	130 (97)	119 (96)			
*No*	143 (100)	4 (3)	7 (6)			
Hypotensive drugs						7.85E-07
*Yes*	0 (0)	40 (30)	76 (60)			
*No*	143 (100)	94 (70)	50 (40)			
Duration of T2DM (years)	NA	10.7 ± 7.7	10.5 ± 6.1			0.826
Family history						
*No*	143 (100)	65 (49)	64 (51)			0.713
*T2DM*	0 (0)	55 (41)	10 (8)			7.21E-10
*Hypertension*	0 (0)	3 (2)	13 (10)			0.007
*CHD*	0 (0)	1 (1)	14 (11)			3.41E-04
*T2DM, hypertension*	0 (0)	7 (5)	11 (9)			0.266
*T2DM, CHD*	0 (0)	0 (0)	4 (3)			0.038
*Hypertension, CHD*	0 (0)	3 (2)	7 (6)			0.165
*T2DM, hypertension, CHD*	0 (0)	0 (0)	3 (2)			0.072

Data was presented as mean ± SD or participant numbers (%), unless otherwise specified. P values were calculated by hypothesis testing. For continuous variables, the distribution of the variable was first assessed by the Shapiro–Wilk test. Bilateral student’s t-test was used for normally distributed data. While the Mann–Whitney U test was used for non-parametric data. For categorical variables, P values were calculated using the Chi-square test.

### Results of clinical laboratory test

A total of 47 clinical indicators were obtained by clinical laboratory test, which were mainly involved in the blood biochemistry, blood pressure, erythrocyte function, blood platelet, blood coagulation function, and thyroid function. As shown in Table 2, clinical indicators varied widely among the HC, T2DM and T2DM-CHD groups. Blood lipid indicators, including total cholesterol (TC) and low density lipoprotein (LDL-C), were significantly lower in the T2DM-CHD group compared to the T2DM group. High density lipoprotein (HDL-C), known as a lipid scavenger and a protective factor for CHD, was also significantly lower in the T2DM-CHD groups than the T2DM groups. Direct bilirubin (DBIL) and indirect bilirubin (IBIL) showed an opposite trend, with DBIL significantly higher and IBIL significantly lower in the T2DM-CHD group compared to the T2DM group.

**TABLE 2:** Results of clinical laboratory test.

Characteristics	HC	T2DM	T2DM-CHD	*P*_1_ value (HC *vs* T2DM)	*P*_2_ value (HC *vs* T2DM-CHD)	*P*_3_ value (T2DM *vs* T2DM-CHD)
**Blood biochemistry**						
TC (mmol/L)	4.56 ± 0.94	4.73 ± 1.06	3.93 ± 1.07	0.134	7.58E-07^&&^	6.00E-09**
HDL-C (mmol/L)	1.33 ± 0.34	1.25 ± 0.40	1.09 ± 0.29	0.010	3.10E-09^&&^	0.001**
LDL-C (mmol/L)	2.62 ± 0.72	2.82 ± 0.84	2.20 ± 0.84	0.028	1.65E-05^&&^	1.06E-08**
TG (mmol/L)	1.32 ± 0.69	1.70 ± 1.17	1.68 ± 1.20	0.005	0.025^&^	0.618
Glucose (mmol/L)	5.24 ± 2.17	8.50 ± 3.68	9.51 ± 4.49	1.15E-22	9.22E-26^&&^	0.111
Uric (μmol/L)	303 ± 71	315 ± 78	334 ± 91	0.212	0.006^&&^	0.120
DBIL (μmol/L)	3.47 ± 1.86	3.23 ± 2.32	4.03 ± 2.10	0.163	0.0342^&^	7.00E-04**
IBIL (μmol/L)	9.44 ± 4.20	10.3 ± 5.80	8.74 ± 4.39	0.493	0.1090	0.041*
TBA (μmol/L)	4.44 ± 3.68	4.06 ± 3.78	4.10 ± 4.28	0.262	0.1338	0.616
Creatinine (μmol/L)	70.6 ± 16.8	70.2 ± 16.5	77.7 ± 21.9	0.514	0.0248^&^	0.008**
HsCRP (mg/L)	NA	2.93 ± 3.27	4.24 ± 3.82			0.001**
AST (U/L)	24.1 ± 10.5	28.0 ± 71.3	69.6 ± 111.8	0.006	0.057	2.10E-04**
ALT (U/L)	21.6 ± 11.1	25.4 ± 25.3	32.8 ± 35	0.579	9.09E-05^&&^	0.002**
CK (U/L)	98.6 ± 45.1	90.7 ± 51.0	442 ± 877	0.013	0.240	0.005**
MMB (ng/L)	NA	NA	32.7 ± 74.3			
CTNI (ng/L)	NA	NA	16.3 ± 37.3			
LDH (U/L)	172 ± 29	158 ± 40	282 ± 258	2.88E-05	0.005^&&^	3.08E-08**
HBDH (U/L)	140 ± 23	134 ± 27	252 ± 252	0.030	1.37E-04^&&^	3.93E-07**
ALP (U/L)	82.7 ± 23.7	91.5 ± 25.1	86.6 ± 26.5	7.06E-04	0.143	0.067
GGT (U/L)	30.1 ± 26.2	40.2 ± 52.0	36.8 ± 31.4	0.083	0.007^&&^	0.582
HCY (μmol/L)	NA	13.5 ± 4.8	16.9 ± 6.3			1.57E-07**
NT-proBNP (pg/mL)	NA	NA	1,146 ± 2,098			
HbA1c (%)	NA	9.85 ± 2.23	8 ± 1.61			3.55E-10**
**Blood pressure**						
SBP (mmHg)	128 ± 16	146 ± 26	158 ± 30	1.97E-10	9.00E-18^&&^	2.56E-04**
DBP (mmHg)	78.4 ± 10.9	83.9 ± 15.3	90.7 ± 20.1	3.52E-04	1.96E-08^&&^	0.006**
**Erythrocyte function**						
RBC (× 10^12^/L)	4.58 ± 0.47	4.47 ± 0.48	4.36 ± 0.54	0.094	0.001^&&^	0.071
HGB (g/L)	142 ± 13	138 ± 16	140 ± 92	0.018	7.26E-06^&&^	0.030*
HCT (%)	41.4 ± 3.5	39.6 ± 4.2	38.6 ± 4.6	2.38E-04	1.16E-07^&&^	0.068
MCV (fl)	90.7 ± 4.6	88.8 ± 5.2	88.7 ± 4.8	4.05E-04	0.002^&&^	0.773
MCH (pg)	31.1 ± 2.1	30.8 ± 2.2	30.3 ± 2.1	0.443	0.015^&^	0.077
MCHC (g/L)	343 ± 11	346 ± 12	343 ± 12	0.003	0.990	0.005**
RDW-CV (%)	12.9 ± 0.9	12.8 ± 1.0	13.1 ± 1.3	0.090	0.036^&^	3.56E-04**
**Blood platelet**						
PLT (× 10^9^/L)	213 ± 56	219 ± 53	207 ± 49	0.239	0.483	0.043*
PDW (fl)	12.6 ± 2.2	12.6 ± 1.8	12.4 ± 1.7	0.494	0.833	0.352
MPV (fl)	10.5 ± 1	10.5 ± 0.9	10.6 ± 0.9	0.605	0.197	0.371
P-LCR (%)	28.8 ± 8.2	29.1 ± 7.1	29.6 ± 7.1	0.534	0.254	0.547
**Coagulation function**						
PT (s)	11.3 ± 0.8	11.2 ± 0.8	11.5 ± 1.9	0.094	0.837	0.228
PA (%)	93.7 ± 6.3	97.8 ± 8	93.9 ± 10.6	4.03E-06	0.707	4.36E-04**
PR	1.00 ± 0.06	0.98 ± 0.07	1.01 ± 0.15	0.001	0.461	0.028*
INR	0.99 ± 0.05	0.96 ± 0.06	0.99 ± 0.16	1.45E-05	0.573	0.001**
APTT (s)	28.6 ± 3.5	27.6 ± 3.9	28.8 ± 5.6	0.020	0.457	0.247
FBG (mg/dL)	263 ± 51	292 ± 72	296 ± 75	1.82E-04	9.47E-05^&&^	0.712
TT (s)	19.1 ± 1.5	18.7 ± 1.7	20.0 ± 9.3	0.004	0.354	0.094
D-DIMER (mg/L)	NA	0.36 ± 0.50	0.42 ± 0.57			0.016*
**Thyroid Function**						
FT3 (pg/mL)	NA	2.61 ± 0.43	2.58 ± 0.75			0.124
FT4 (ng/dl)	NA	1.05 ± 0.14	1.06 ± 0.17			0.571
STSH (uIU/ml)	NA	1.91 ± 1.97	1.66 ± 1.62			0.079

Data was presented as mean ± SD, unless otherwise specified. P values were calculated by hypothesis testing. Data distribution was assessed by the Shapiro–Wilk test. Bilateral student’s t-test was used for normally distributed data. While the Mann–Whitney U test was used for non-parametric data. (&, *), P < 0.05; (&&, **), P < 0.01.

Enzyme and protein indicators associated with myocardial injury, including HsCRP, AST, ALT, CK, LDH, and hydroxybutyrate dehydrogenase (HBDH) were all significantly higher in the T2DM-CHD group than the T2DM group. Homocysteine (HCY), a risk factor of cardiovascular disease, was significantly elevated in the T2DM-CHD group as expected. Blood pressure in the T2DM group was significantly higher than that in the other groups. Blood cell related indicators, including hemoglobin (HGB), mean corpuscular hemoglobin concentration (MCHC), red blood cell distribution width (RDW-CV), and platelets (PLT) were significantly different between T2DM and T2DM-CHD groups. Coagulation function related indicators, including prothrombin activity (PA), prothrombin time ratio (PR), international normalized ratio (INR), and D-dimer, were significantly different between T2DM and T2DM-CHD groups. No significant differences were observed in thyroid-related indicators between the T2DM and T2DM-CHD groups.

### Free fatty acids levels in plasma

**FIGURE 1:**
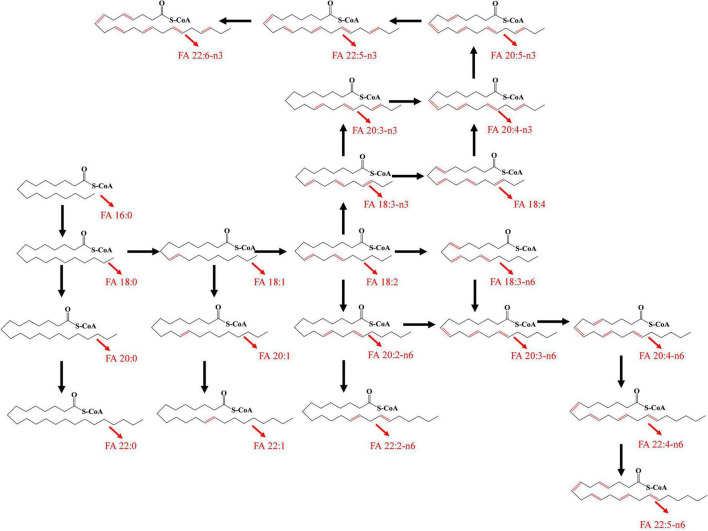
Biosynthetic pathway of unsaturated FFAs.

**FIGURE 2:**
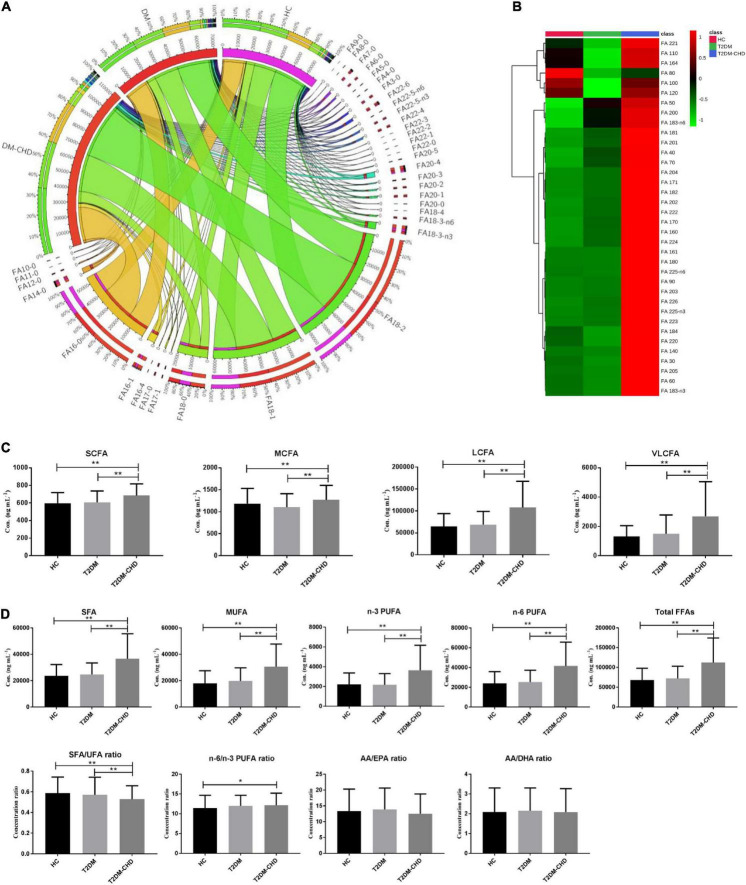
**(A)** Circos plot exhibited the distribution of FFAs in each groups. **(B)** Heatmap showing the average FFA concentrations in each group. **(C)** Histogram of FFAs with different carbon chain lengths. **(D)** Histogram of FFAs with different unsaturation. The ratios of different FFAs were also compared. *P < 0.05; **P < 0.01. *P* values were calculated by hypothesis testing. Data distribution was assessed by the Shapiro–Wilk test. Bilateral student’s *t*-test was used for normally distributed data. While the Mann–Whitney *U* test was used for non-parametric data.

A total of 36 FFAs with carbon chain length ranged from 3 to 22 were detected and quantified in plasma samples. Biosynthetic pathway of both n-3 and n-6 unsaturated FFAs was shown in Figure 1. A Circos plot exhibited the distribution of FFAs in each group was shown in Figure 2A. Linoleic acid (FA 18:2) was the most abundant FFA in plasma, followed by oleic acid (FA 18:1), palmitic acid (FA 16:0), and stearic acid (FA 18:0). Linoleic acid is an omega-6 unsaturated FFAs, which occurs widely in organism. It is an essential FFA for human, because it cannot be synthesized by the organism. As shown in Figure 1, linoleic acid was the precursor of the both n-3 and n-6 polyunsaturated FFAs. It can also be converted to the inflammation-associated lipids such as prostaglandins, leukotrienes, and thromboxane. Other FFAs with high abundance in plasma include palmitoleic acid (FA 16:1), alpha linolenic acid (FA 18:3-n3), arachidonic acid (FA 20:4), and docosahexaenoic acid (FA 22:6). The mean concentrations of FFAs in each group were listed in Table 3. The average concentrations of each FFA in the HC, T2DM and T2DM-CHD groups were shown in a heatmap (Figure 2B). As shown in Table 3 and Figure 2A, the levels of almost all FFAs in the T2DM-CHD group were significantly higher than that of the T2DM group.

**TABLE 3:** FA concentrations (mean ± SD) in different groups.

FFAs	Abbreviation	Classification	HC (ng/mL)	T2DM (ng/mL)	T2DM-CHD (ng/mL)	*P*_1_ value (HC *vs* T2DM)	*P*_2_ value (HC *vs* T2DM-CHD)	*P*_3_ value (T2DM *vs* T2DM-CHD)
Propionic acid	FA 3:0		412.6 ± 107.8	408.0 ± 105.0	461.8 ± 113.8	0.673	2.90E-04^&&^	4.52E-05**
Butyric acid	FA 4:0		96.9 ± 16.5	101.6 ± 39.6	117.6 ± 29.9	0.400	1.01E-11^&&^	2.59E-09**
Pentanoic acid	FA 5:0		87.9 ± 22.0	98.4 ± 27.1	106.0 ± 28.0	0.001	3.71E-08^&&^	0.017*
Caproic acid	FA 6:0		363.1 ± 76.2	359.4 ± 81.4	412.3 ± 91.7	0.396	7.80E-06^&&^	7.56E-07**
Heptanoic acid	FA 7:0		477.2 ± 169.6	483.6 ± 172.8	508.7 ± 196.8	0.859	0.278	0.437
Octanoic acid	FA 8:0		36.6 ± 19.9	34.0 ± 30.0	34.8 ± 12.5	0.050	0.997	0.056
Non-anoic acid	FA 9:0		10.0 ± 3.2	10.0 ± 6.0	10.8 ± 4.1	0.060	0.102	0.003**
Decanoic acid	FA 10:0		72.0 ± 63.1	51.3 ± 70.0	69.6 ± 54.8	4.62E-05	0.771	1.56E-05**
Undecanoic acid	FA 11:0		2.9 ± 1.6	2.5 ± 1.8	3.2 ± 2.1	0.008	0.097	1.62E-05**
Lauric Acid	FA 12:0		223.7 ± 190.8	164.6 ± 108.2	231.4 ± 147.0	0.003	0.155	3.02E-05**
Myristic Acid	FA 14:0		858.9 ± 553.5	825.3 ± 533.3	1,693.7 ± 1,522.7	0.999	7.65E-08^&&^	3.09E-08**
4Z,7Z,10Z,13Z-Hexadecatetraenoic Acid	FA 16:4	n3	47.7 ± 32.9	41.1 ± 37.6	52.9 ± 36.8	0.016	0.186	2.72E-04**
Palmitoleic Acid	FA 16:1		1,322.6 ± 962.0	1,314.8 ± 977.5	2,705.6 ± 2,727.8	0.745	1.14E-06^&&^	1.16E-06**
Palmitic Acid	FA 16:0		14,566.1 ± 6,108.5	15,656.6 ± 6,297.9	24,295.0 ± 13,625.1	0.137	9.42E-10^&&^	5.31E-07**
*cis-*10-Heptadecenoic Acid	FA 17:1		104.6 ± 71.4	114.7 ± 81.0	248.5 ± 269.0	0.270	6.31E-10^&&^	1.04E-07**
Heptadecanoic Acid	FA 17:0		198.8 ± 102.8	225.3 ± 120.3	419.4 ± 355.7	0.039	1.07E-11^&&^	7.71E-08**
Stearidonic Acid	FA 18:4	n3	16.2 ± 15.6	13.4 ± 12.9	33.3 ± 50.5	0.070	2.45E-06^&&^	8.95E-10**
α-Linolenic Acid	FA 18:3-n3	n3	1,341.4 ± 759.0	1,294.8 ± 700.2	2,001.5 ± 1,284.2	0.820	7.69E-06^&&^	2.28E-06**
γ-Linolenic Acid	FA 18:3-n6	n6	188.2 ± 114.2	305.8 ± 1,061.2	452.8 ± 538.2	0.939	5.35E-10^&&^	9.57E-10**
Linoleic Acid	FA 18:2	n6	22,130.2 ± 10,977.1	23,278.3 ± 11,118.4	37,945.4 ± 21,482.2	0.358	1.52E-09^&&^	5.56E-08**
Oleic Acid	FA 18:1		16,218 ± 8,467.2	18,019.8 ± 8,781.8	26,990.7 ± 14,207.2	0.073	2.30E-10^&&^	3.50E-07**
Stearic Acid	FA 18:0		6,227.1 ± 1,965.1	6,208.8 ± 1,937.8	8,202.2 ± 3,657.2	0.868	5.11E-06^&&^	2.00E-06**
Eicosapentaenoic Acid (EPA)	FA 20:5	n3	85.4 ± 80.6	80.7 ± 65.9	164.1 ± 224.4	0.689	4.29E-05^&&^	6.07E-05**
Arachidonic Acid (AA)	FA 20:4	n6	910.5 ± 599.0	960.0 ± 601.4	1,547.9 ± 1,391.5	0.380	2.54E-05^&&^	4.54E-04**
Dihomo-γ-Linolenic Acid	FA 20:3	n6	145.5 ± 100.5	149.0 ± 126.9	331.2 ± 351.0	0.751	2.58E-08^&&^	8.03E-08**
8Z,14Z-Eicosadienoic Acid	FA 20:2	n6	233.3 ± 120.7	259.6 ± 136.0	487.1 ± 384.6	0.085	1.10E-10^&&^	1.70E-07**
11E-Eicosenoic Acid	FA 20:1		301.2 ± 176.0	349.7 ± 204.8	626.6 ± 551.5	0.034	3.90E-10^&&^	2.34E-06**
Arachidic Acid	FA 20:0		49.8 ± 18.1	66.7 ± 64.3	88.5 ± 86.7	0.302	1.08E-06^&&^	1.36E-04**
Docosahexaenoic Acid (DHA)	FA 22:6	n3	468.0 ± 261.9	484.9 ± 255.9	775.5 ± 564.0	0.308	3.31E-07^&&^	1.29E-05**
Docosapentaenoic Acid	FA 22:5-n3	n3	244.7 ± 179.2	259.1 ± 180.4	605.9 ± 672.2	0.210	8.29E-10^&&^	7.43E-08**
4,7,10,13,16-Docosapentaenoic Acid	FA 22:5-n6	n6	77.3 ± 53.4	76.5 ± 46.5	139.7 ± 133.0	0.509	1.86E-06^&&^	1.29E-05**
7Z,10Z,13Z,16Z-Docosatetraenoic Acid	FA 22:4	n6	279.6 ± 175.5	319.0 ± 194.6	631.8 ± 610.9	0.042	1.50E-11^&&^	1.88E-07**
Docosatrienoic Acid	FA 22:3	n3	6.7 ± 2.7	6.8 ± 3.5	11.5 ± 7.6	0.829	1.09E-09^&&^	2.42E-09**
13Z,16Z-Docosadienoic Acid	FA 22:2	n6	9.3 ± 4.3	10.0 ± 4.8	16.4 ± 11.4	0.284	5.84E-09^&&^	7.82E-07**
13Z-Docosenoic Acid	FA 22:1		37.1 ± 45.4	32.1 ± 23.2	45.8 ± 39.6	0.923	7.94E-04^&&^	4.15E-04**
Docosanoic Acid	FA 22:0		7.8 ± 6.6	7.3 ± 3.4	11.0 ± 12.0	0.685	2.86E-04^&&^	7.74E-05**

Data was presented as mean ± SD, unless otherwise specified. P values were calculated by hypothesis testing. Data distribution was assessed by the Shapiro–Wilk test. Bilateral student’s t-test was used for normally distributed data. While the Mann–Whitney U test was used for non-parametric data. (&, *), P < 0.05; (&&, **), P < 0.01.

FFAs can be divided into short chain fatty acid (SCFA, < 6C), medium chain fatty acid (MCFA, 6-12C), long chain fatty acid (LCFA, 12-20C), and very long chain fatty acid (VLCFA, ≥ 22C) depending on the carbon chain length. As shown in Figure 2C, T2DM-CHD possessed the highest concentrations of SCFA, MCFA, LCFA and VLCFA among the three groups. FFAs of different saturations were also statistically analyzed. As shown in Figure 2D, the concentration levels of saturated fatty acid (SFA), monounsaturated fatty acid (MUFA), polyunsaturated fatty acids (PUFA), and total FFAs in the T2DM-CHD groups were all significantly higher than those in the HC or T2DM groups. The ratio between SFA and unsaturated FFAs (UFA) were investigated with result shown in Figure 2D. The disease groups had lower SFA/UFA ratios than the HC group, and T2DM-CHD group had the lowest SFA/UFA ratio, which was significantly lower than that of T2DM group and HC group. The ratio of n-6 and n-3 PUFAs were also compared (Figure 2D). No significantly differences of the n-6/n-3 PUFA ratio, arachidonic acid/eicosapentaenoic acid (AA/EPA) ratio, or arachidonic acid/docosahexaenoic acid (AA/DHA) ratio were observed between T2DM and T2DM-CHD groups.

### Correlation analysis of the clinical indicators and free fatty acids

**FIGURE 3:**
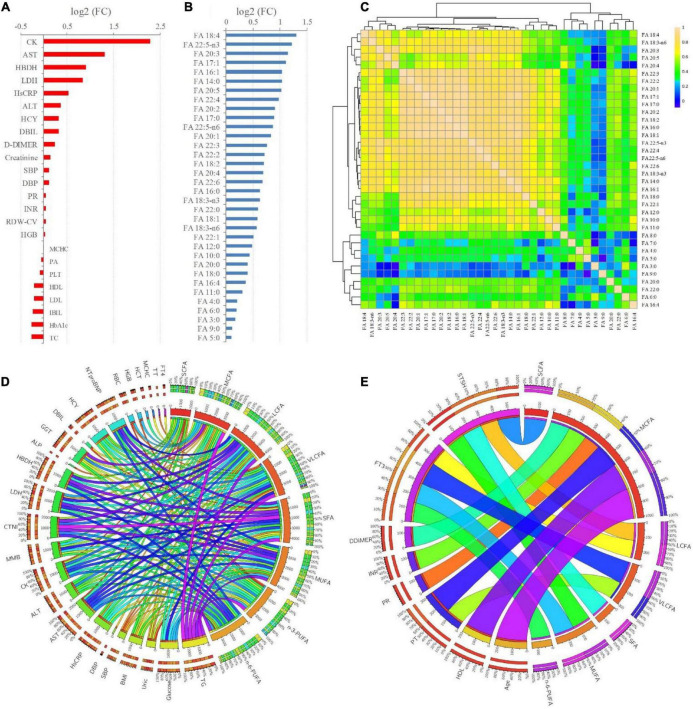
**(A,B)** FCs (T2DM-CHD/T2DM) of the significantly changed (*P* < 0.05) clinical indicators and FFAs existed between T2DM and T2DM-CHD groups, respectively. **(C)** Spearman’s rank correlation analysis of the FFAs. **(D,E)** Significant positive and negative correlations (*P* < 0.05) existed between FFAs and clinical indictors, respectively.

Fold changes (FCs) of all the significantly changed clinical indictors and FFAs between the T2DM and T2DM-CHD groups were shown in Figures 3A,B, respectively. All the clinical indictors and FFAs presented higher levels in the T2DM-CHD group than the T2DM group, except for MCHC, PA, PLT, HDL-C, LDL-C, IBIL, HbA1c, and TC. Indicators with FCs greater than 2 included CK, AST, FA 18:4, FA 22:5-n3, FA 20:3, FA 17:1, FA 16:1, FA 14:0, and FA 20:5.

Since the organism presents a complex network, a correlation analysis can well reflect the internal relationship between FAs and clinical key indicators. The correlations among each of the FFA were first explored based on Spearman’s rank correlation analysis. As presented in Figure 3C, significant positive correlations were existed among the FFAs, especially the LCFAs and VLCFAs. Further, Spearman’s rank correlation analysis was conducted between FFAs and clinical indicators. Only significant positive and negative correlations (*P* < 0.05) were displayed in Figures 3D,E, respectively. There were significantly positive correlations between TG, glucose, HsCRP, AST, ALT, CK, MMB, CTNI, LDH, HBDH, GGT, HCY, NTproBNP and FFAs (Figure 3D). The thyroid function related indicators FT3 and STSH were negatively correlated with FFAs (Figure 3E). Besides, coagulation function related indicators of PT, PR, INR, DDIMER, and thyroid function related indicators of FT3 and STSH were negatively correlated with FFAs (Figure 3E).

### Potential biomarkers for the distinguish of T2DM-CHD patients

**FIGURE 4:**
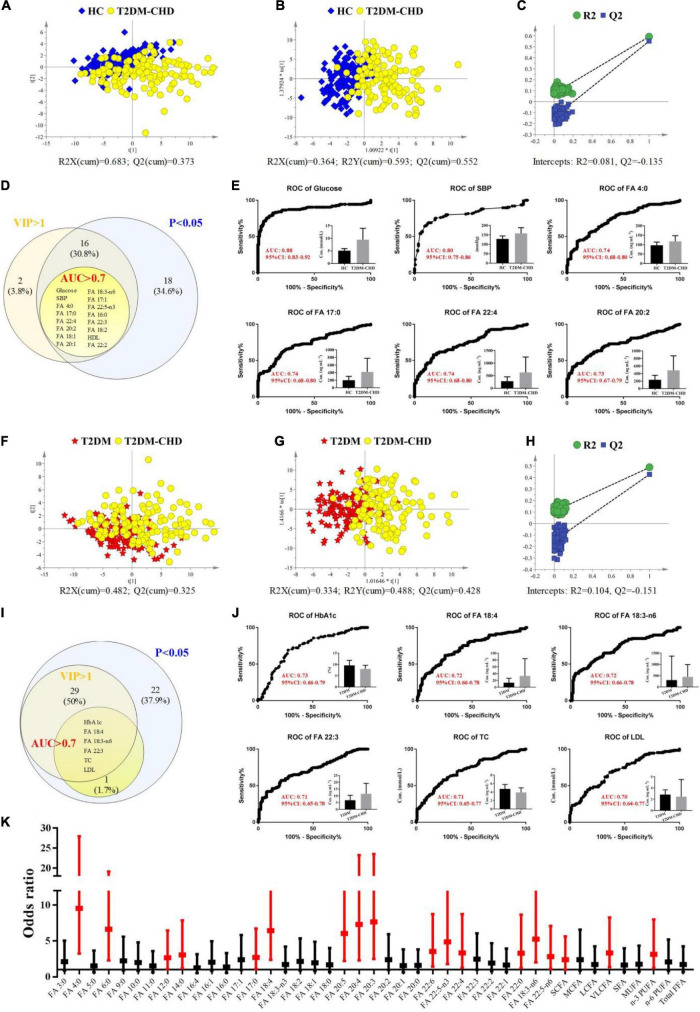
**(A–E)** Exploration of differences between HC and T2DM-CHD groups. **(A)** PCA score scatter plot. **(B)** OPLS-DA score scatter plot. **(C)** Random permutation test of the OPLS-DA model. **(D)** Venn diagram depicting the overlap of different statistical methods. **(E)** ROC curves of the potential biomarkers for the discrimination of HC and T2DM-CHD groups. **(F–J)** Exploration of differences between T2DM and T2DM-CHD groups. **(F)** PCA score scatter plot. **(G)** OPLS-DA score scatter plot. **(H)** Random permutation test of the OPLS-DA model. **(I)** Venn diagram depicting the overlap of different statistical methods. **(J)** ROC curves of the potential biomarkers for the discrimination of T2DM and T2DM-CHD groups. **(K)** A forest plot of the significantly changed FFAs between T2DM and T2DM-CHD groups. Red indicates FFAs with *P* < 0.05 in multivariate analysis.

Multivariate statistical analysis was conducted based on clinical indicators and FFAs levels to screen potential biomarkers that can be used in the diagnosis of CHD patients with a T2DM history. Both unsupervised PCA and supervised OPLS-DA model were development for the discrimination of T2DM-CHD group and the other two groups. As shown in Figures 4A,B, separation of the T2DM-CHD and HC groups can be achieved on both PCA and OPLS-DA score plots. No overfitting of the OPLS-DA model was observed through a random permutation test with 100 iterations Figure 4C. To screen potential biomarkers associated with the diagnosis of T2DM-CHD, the variable importance in the projection (VIP) of each metabolite was calculated based on the established OPLS-DA model. Finally, a total of 34 indexes, including 9 clinical indicators and 25 FFAs, were found to possessed VIP values larger than 1. A Venn diagram depicting the overlap of different statistical methods was shown in Figure 4D. Sixteen indexes, including glucose, SPB, HDL-C and 13 FFAs, were found to simultaneously satisfy the conditions of VIP > 1, *P* < 0.05, and area under (AUC) the receiver operating characteristic (ROC) curve larger than 0.7. These 16 indexes were screened as potential biomarkers for the discrimination of HC and T2DM-CHD patients. The ROC curves of 6 indexes with the largest AUC values were shown in Figure 4E.

The same multivariate statistical methods were used to analyze and compare the differences between the T2DM-CHD group and the T2DM group, with results shown in Figures 4F–H. Moderate separation of the two groups can be achieved in both PCA and OPLS-DA models, with no overfitting of the OPLS-DA model observed. Finally, seven indexes, including HbA1c, TC, LDL-C, FA 18:4, FA 18:3-n6, and FA 22:3, were found to satisfy the conditions of VIP > 1, *P* < 0.05, and AUC > 0.7 (Figure 4I). The ROC curves of 6 indexes with the largest AUC values were shown in Figure 4J, which were screened as potential biomarkers for the distinguish of T2DM-CHD patients from the T2DM patients.

To further investigate whether high FFAs were risk factors independent of the tradition risk factors of cardiovascular disease (i.e., HsCRP, HCY, CK et al.), the concentration values of FFAs with significant differences between T2DM-CHD and T2DM groups were converted to ordered binary variables, with the minimum 50% defined as low level, while the maximum 50% defined as high level. The transformed ordered binary variable of each FFA, together with the BMI, age, smoking and drinking history, and all the significantly changed clinical indicators listed in Table 2, were submitted to multivariate logistic regression analysis. Odds ratio (OR) values and the corresponding 95% confidence interval (CI) of all the FFAs after adjustment for other confounding factors were shown in Figure 4K in the form of a forest plot. A dozens of FFAs were found to be independent risk factors of CHD in patients with T2DM.

## Discussion

A variety of studies have reported that the elevated FFA levels in plasma were associated with both the presence and severity of cardiovascular events in patients with T2DM (29–31). However, these previous studies mainly focused on the total FFA levels in plasma, and few of them paid attention to the fingerprint of FFAs. In this study, we interpreted the fingerprint of plasma FFAs in T2DM patients with CHD, and explored their concentrations differences from the healthy controls and the T2DM patients without CVD. A total of thirty-six FFAs with different carbon chain length (3–22 carbons) and different saturations (0–6 double bonds) were analyzed by using UHPLC-MS/MS. All these 36 FFAs can be successfully detected and quantified in plasma samples. In addition, the relationships between plasma FFAs and cardiac enzymes, glucose, clotting and thyroid functions were also investigated in this study.

The mean BMI value of the T2DM-CHD group was slightly higher than that of the T2DM group, but the difference was not statistically significant. Blood lipid indicators, including TC and LDL-C, were significantly lower in the T2DM-CHD group than that in the T2DM group, which was contrary to the trend of BMI (Table 2). TC is the total amount of cholesterol in human blood. Low-density lipoprotein particles are the main carriers of cholesterol, each containing about 1,500 cholesterol ester molecules. Blood test commonly report LDL-C, which is the amount of cholesterol estimated to be contained with low-density lipoprotein. LDL-C is often called the “bad” cholesterol because it sticks to the walls of blood vessels, raising the chances of health problems such as heart attack and stroke. The opposite trend of BMI and cholesterol levels existed between T2DM-CHD and T2DM groups was believed to be related to the medication of lipid-lowering drugs. More subjects in the T2DM-CHD group were receiving lipid-lowering medications than those in the T2DM groups (Table 1). A significant negative correlation (*r* = −0.166, *P* = 0.008) was showed between the medication of lipid-lowering drugs and the blood levels of LDL-C. Besides the lower TC and LDL-C levels in the T2DM-CHD group, no significant differences of TG was observed between T2DM-CHD and T2DM groups. HDL-C is known as the “good” cholesterol because it helps remove other forms of cholesterol from the bloodstream. Higher blood level of HDL-C is associated with a lower risk of heart disease. HDL-C level is typically lower in people who have metabolic syndrome of obesity, high blood pressure and high blood glucose levels, which was consistent with the results of this study.

FFAs are non-esterified fatty acids circulating in the circulatory system by bounding to the albumin. FFAs are the main energy source of myocardial tissue. Release of FFAs from triglyceride and phospholipids is regulated by the action of insulin and modulated by adrenergic activity. FFA metabolism will also be disturbed under the condition of insulin dysfunction. FFA levels are usually elevated in obese people, which contribute to T2DM and cardiovascular disease. The elevated FFAs will in turn promote the occurrence of insulin resistance. In this study, almost all the FFAs were significantly higher in the T2DM-CHD group among the three different groups (Table 2). Especially for LCFAs and VLCFAs, their concentration levels of T2DM-CHD groups were generally more than 1.5 fold higher than that of the T2DM group (Figure 3B). Despite of the significantly lower blood lipid indicators of TC and LDL-C in T2DM-CHD group, almost all the FFAs were increased under the condition of CHD. Unlike the cholesterol, lipid-lowering drugs did not significantly reduce the FFA levels of patients in the CHD group (Table 2). Plasma FFAs levels were significantly higher in the T2DM-CHD groups than the T2DM group. The metabolism of FFAs may become deleterious *via* mechanisms such as oxidative stress and arrhythmogenesis under certain conditions such as heart failure or myocardial ischemia.

Omega-3 PUFAs, mainly including EPA and DHA, have shown promise for the prevention of cardiovascular disease and cardiovascular death. Numerous epidemiological and clinical studies showed that the supplementation of n-3 PUFAs is associated with decreased inflammation (32, 33). N-3 PUFAs usually exhibited lower levels in patients with cardiovascular disease (33). However, there are some differences in our results. The n-3 PUFA presented higher concentrations in the subjects with CHD than the subjects without CHD (Table 2 and Figure 2). No significant differences of n-6/n-3 FFA ratios were observed between T2DM-CHD and T2DM groups. One large randomized controlled trial (*n* = 12,536, 6.2 years follow-up) designed to examine the effects of n-3 PUFA supplementation on cardiovascular death among patients with or at risk of diabetes mellitus do not support n-3 PUFA supplementation among patients with diabetes mellitus or prediabetes to prevent cardiovascular events (34). Another large randomized controlled trial (*n* = 25,871, 5.3 years follow-up) revealed that the supplementation with n-3 PUFA did not result in a lower incidence of major cardiovascular events among men 50 years of age or older and women 55 years of age or older (35). A review of large randomized controlled trial by David S. Siscovick do not recommend n-3 PUFA treatment for patients with diabetes mellitus and prediabetes to prevent CHD (36). There was a lack of consensus on the recommendation for patients at high cardiovascular disease risk (36).

Clinical indicators related to the blood biochemistry, blood pressure, erythrocyte function, and blood coagulation function were extensively disturbed in the T2DM-CHD patients, which was significantly different from the HC and T2DM groups (Table 2). Elevated HsCRP, HCY, AST, ALT, CK, MMB, CTNI, LDH, HBDH, and NT-proBNP are traditional risk factors of cardiovascular disease. High HsCRP levels in the blood is a marker of inflammation in the arteries of the heart, which can increase likelihood for heart attack or stroke. A high HCY levels, also called hyperhomocysteinemia, can contribute to arterial damage and blood clots in the blood vessels. CK, CTNI, AST, ALT, MMB, LDH and HBDH are called the cardiac enzymes or cardiac biomarkers. They are proteins from heart muscle cells that have leaked out into the bloodstream after an injury to the cardiac muscle. NT-proBNP is cardiac hormones secreted by ventricular myocardium in response to the increased pressure inside the heart, which is a specific biomarkers of heart failure and other cardiac problems. Significant elevated HsCRP, HCY, AST, ALT, CK, MMB, CTNI, LDH, HBDH, and NT-proBNP in the T2DM-CHD patients indicated the occurrence of myocardial damage. Increased D-dimer is indicative of a hypercoagulable state, which was found to be associated with acute coronary syndromes (37–39). Actually, the concentrations of D-dimer were demonstrated to remain elevated for several months after AMI (40). In this study, plasma D-dimer level in T2DM-CHD group was higher than that in T2DM group (Table 2), which was caused by hypercoagulability state in the background of cardiovascular disease.

Plasma FFAs of different carbon chain length and different saturations exhibited significant positive correlations with these traditional risk factors of cardiovascular disease (Figure 3D). Multivariate logistic regression analysis demonstrated that the increased FFA levels were independent risk factors of coronary heart disease after adjusting for these traditional cardiac biomarkers (Figure 4K). Clinical study of large sample size demonstrated that the elevated FFA levels are a strong independent predictor of cardiac death and other cardiovascular deaths after multivariable adjustment for a broad range of other potential risk factors (41, 42).

Despite these findings, several limitations needed to be disclosed in this study. First, this is a cross-sectional study. Plasma FFA fingerprints during progression from T2DM to T2DM-CHD could not be traced. The plasma levels of some FFAs is usually affected by nutritional status, it may not be able to accurately reflect the change of plasma FFAs with the development of CHD in patients with T2DM. Second, the participants involved in this study were healthy controls or patients suffering T2DM. We were not able to study FFA differences of CHD patients with and without T2DM. Third, this is a pilot study of single center study and small sample size. The plasma fingerprints associated with T2DM-CHD need to be validated with larger sample sizes and a wider range of populations.

## Conclusion

In conclusion, this study exhibited the plasma fingerprint of FFAs and their correlations with the traditional cardiac biomarkers in CHD patients with T2DM. Almost all the FFAs with different carbon chain length and unsaturation presented significantly higher plasma levels in the CHD-T2DM patients than patients with only T2DM. However, the ratio of n-6/n-3 PUFA did not change significantly between the CHD-T2DM and CHD groups. Significantly positive relationships were existed between FFA concentrations and the traditional risk factors of CHD. A dozens of FFAs were found to be the independent risk factors of CHD in people with T2DM after adjustment for the tradition risk factors of CHD and other confounding factors.

## Data availability statement

The original contributions presented in the study are included in the article/supplementary material, further inquiries can be directed to the corresponding author/s.

## Ethics statement

The studies involving human participants were reviewed and approved by the Ethics Committees of Beijing Chao-Yang Hospital. Written informed consent for participation was not required for this study in accordance with the national legislation and the institutional requirements.

## Author contributions

TH, ZA, and LL conceived and designed this study. TH and WZ performed the experiments and wrote and revised the manuscript. WZ and FH determined the clinical phenotype. WZ and RZ collected the clinical samples. TH interpreted the data. All authors contributed to the article and approved the submitted version.
